# Effect of body mass index (BMI) on phenotypic features of polycystic ovary syndrome (PCOS) in Singapore women: a prospective cross-sectional study

**DOI:** 10.1186/s12905-021-01277-6

**Published:** 2021-04-01

**Authors:** Samantha A. Neubronner, Inthrani R. Indran, Yiong Huak Chan, Angelica Win Pa Thu, Eu-Leong Yong

**Affiliations:** 1 Department of Obstetrics and Gynaecology, Yong Loo Lin School of Medicine, National University of Singapore, NUHS Tower Block, 1E Kent Ridge Road, Level 12, Singapore, Republic of Singapore 119228; 2 Department of Pharmacology, Yong Loo Lin School of Medicine, National University of Singapore, Singapore, Republic of Singapore; 3 Biostatistics Unit, Yong Loo Lin School of Medicine, National University of Singapore, Singapore, Republic of Singapore

**Keywords:** BMI, PCOS, Clinical, Hormonal and metabolic characteristics

## Abstract

**Background:**

A diagnosis of Polycystic Ovary Syndrome (PCOS) and its related phenotypic features including increased hair growth can affect a woman’s social and emotional well-being. We aim to determine firstly, if excess body weight affects menstrual cycle length, excessive hair growth and other phenotypic features in healthy women without PCOS and secondly, whether having PCOS exacerbates the effects of high body mass index (BMI).

**Methods:**

A prospective cross-sectional study involving healthy women (21–45 years) recruited at an annual health screen for hospital staff and volunteers from the university community, and PCOS cases referred to tertiary gynecological clinics in Singapore. To dissect the independent and/or combinatorial effects of PCOS and BMI on the phenotypic features, subjects were divided into four categories: non-PCOS (normal BMI), non-PCOS (high BMI), PCOS (normal BMI), and PCOS (high BMI). General linear modelling was performed to compare clinical, ovarian, hormonal and metabolic parameters across these four categories.

**Results:**

Of 389 participants, 134 (34.4%) were classified as PCOS and the remaining 255 (65.6%), as the non-PCOS population. Overall 45.2% of women had high BMI (≥ 23). Compared to non-PCOS subjects, women with PCOS had a higher BMI (mean (SD): 25.14 ± 6.46 vs 23.08 ± 4.36, *p* < 0.001). Women with PCOS and high BMI had increased hair growth with modified Ferriman-Gallwey (mFG) scores that were 2.96-fold higher versus healthy-normal BMI women (mean difference; 1.85, 95% CI 0.80–2.90). Compared to healthy-high BMI women, PCOS women with high BMI had significantly higher mean differences in mFG scores (1.79, 95% CI 0.64–2.93). In PCOS women, having high BMI also significantly increased mFG scores by 1.85-fold (mean difference; 1.82. 95% CI 0.52–3.12). This effect was mirrored by the additive effect of BMI and PCOS on free androgen index. No independent effect of high BMI on rates of oligomenorrhoea, antral follicle count, ovarian volume or serum androgens were observed.

**Conclusions:**

We observed an additive effect of body weight to increase hair growth in women with PCOS. Maximum mFG scores were present in PCOS women with high BMI. Such increases in mFG score may affect the self-esteem of women with PCOS.

## Background

A diagnosis of Polycystic Ovary Syndrome (PCOS) and its related phenotypic features including hirsutism and oligomenorrhea [1] can affect a woman’s social and emotional well-being [2] and physical perception of herself [3], thus causing great distress and leading to a diminished quality of life [4, 5]. In recent decades, obesity has reached epidemic proportions globally [6]. Raised body-mass index (BMI) is a known risk factor for diabetes mellitus, coronary artery disease and strokes [7, 8]. Reproductive problems such as menstrual irregularity and infertility are more prevalent in overweight and obese women [9, 10]. Obesity is also closely associated with PCOS, which affects 6–12% of women of reproductive age [11]. The syndrome is a heterogenous condition characterized by three canonical features; oligomenorrhea/anovulation; hyperandrogenism as demonstrated by elevated serum androgens and/or hirsutism; and polycystic ovarian morphology characterized by abnormally high antral follicle counts (AFC) or increased ovarian volume. The presence of two of these three features is sufficient for a diagnosis of PCOS according to the Rotterdam 2003 criteria [12].

The relationship between high BMI and individual phenotypic features of the Rotterdam criteria that characterize PCOS remains unclear. For example, a meta-analysis among women with PCOS indicates that hirsutism, as measured by modified Ferriman-Gallwey score (mFG), was raised only comparing obese versus overweight women, but not when comparing obese versus normal weight women [13]. Effects of obesity on features such as menstrual cycle length and AFC remain unclear, especially in healthy women. We hypothesize that increased body weight affects these individual phenotypic features, and having PCOS may exacerbate them.

In this study, we examined the effects of BMI on individual clinical, ovarian, hormonal and metabolic features associated with PCOS in women with, and without, the syndrome. The aim was to determine firstly if excess body weight affects these phenotypic features in healthy women without PCOS and, secondly whether having PCOS exacerbates the phenotypic effects of raised BMI.

## Methods

### Study design

**Fig. 1:**
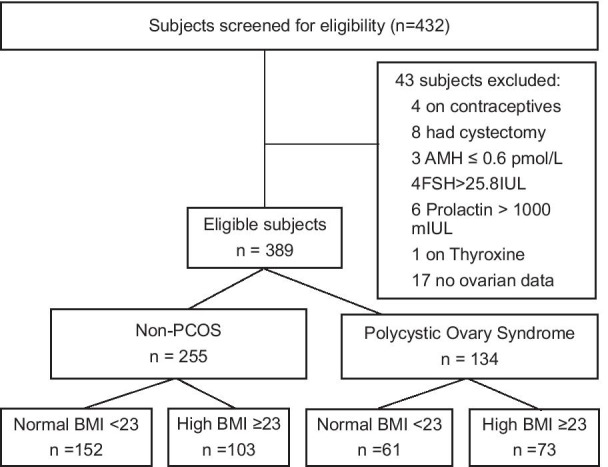
The study population

This is a prospective cohort study involving healthy women and “clinically-suspected” PCOS cases. All eligible participants were assessed similarly, and were classified into two groups: healthy (Non-PCOS) and PCOS (Fig. 1). Details of the protocol have been described previously [14]. Women were diagnosed with PCOS if they presented with at least two out of three features of the Rotterdam criteria [12]. Diagnostic thresholds for AFC (≥ 22), ovarian volume (≥ 8.44 ml) and biochemical hyperandrogenism (serum testosterone ≥ 1.89 nM) have previously been established for this cohort [14]. Hirsutism was defined as mFG score ≥ 5 according to East Asian criteria [15]. Women who were not PCOS served as healthy controls (Fig. 1). The study was approved by the Domain Specific Review Board of the National Healthcare Group.

### Participants

Healthy women, aged 21 to 45 years, were recruited from participants in an annual corporate health screen in National University Hospital (NUH) and volunteers from the university community. “Clinically-suspected” PCOS cases were referred from gynecological clinics at NUH and KK Women’s and Children’s Hospital. Subjects were recruited from 2011 to 2019. Informed written consent was obtained from all participants.

*Exclusion criteria* were pregnancy, breastfeeding, hyperprolactinaemia (previously diagnosed, or with prolactin > 1000 mIU/L), congenital adrenal hyperplasia, adrenal tumours, androgen-secreting tumours, thyroid disease, severe cardiovascular disease, or history of hysterectomy and/or oophorectomy, ovarian failure (anti-müllerian hormone [AMH] levels ≤ 0.6 pmol/L, or follicle stimulating hormone [FSH] levels > 25.8 IUL). Participants on lipid-lowering, and/or contraceptives, diabetic or other medications known to affect reproductive function within 60 days of study entry were also excluded from the study.

### Assessments performed

Eligible subjects completed a demographic survey; reproductive health questionnaire including menstrual cycle profiling and obstetric history; underwent anthropometric evaluation; transvaginal ultrasonography of the ovaries; and blood sampling for reproductive hormones and metabolic biomarkers on days 2 to 5 of the menstrual cycle. Oligomenorrhea was defined as average menstrual cycle length more than or equal to 35 days. Height (m) and Body weight (kg) were measured in a single layer of clothing, without shoes and with pockets emptied. BMI was calculated as Weight/Height^2^. Hair growth was measured using the mFG score [16]. Using reference photographs [17], sexual hair in nine body areas (upper lip, chin, chest, arm, upper abdomen, lower abdomen, upper back, lower back and thighs) were visually scored from one (minimal terminal hairs present) to four (equivalent to a hairy man) by one of two investigators. For standardization of hirsutism scoring, initial cases were independently scored by two investigators and compared till agreement was reached in most cases.

### Variables analyzed

Features of PCOS analyzed included average menstrual cycle length, physical features such as mFG score for hair growth, acne scores and ovarian parameters such as AFC, AMH, and ovarian volume. Laboratory variables measured included testosterone, androstenedione (ADT), dehydroepiandrosterone sulphate (DHEAS), dihydrotestosterone (DHT), sex hormone binding globulin (SHBG), luteinizing hormone (LH), FSH, estradiol, and metabolic variables such as cholesterol, triglycerides, high-density lipoprotein (HDL) and low-density lipoprotein (LDL). Homeostasis model assessment‐estimated insulin resistance (HOMA‐IR), an indicator of insulin resistance, was calculated as (glucose × insulin)/22.5 while free androgen index (FAI) was calculated as total testosterone (nmol/L) × 100/SHBG (nmol/L).

### Statistical analyses

Descriptive analysis for numerical variables were presented as mean ± SD and n (%) for categorical variables. Differences in the numerical variables was performed using independent samples t-test if normality and homogeneity assumptions were satisfied, otherwise the non-parametric Mann Whitney U test will be used. Pearson Chi-square were performed for categorical variables.

Based on the Asia–Pacific classification of BMI [18], non-PCOS and PCOS women were further classified into those with normal BMI < 23 or high BMI ≥ 23 resulting in four categories of subjects (Fig. 1). In order to dissect the independent and/or combinatorial effects of BMI on non-PCOS and PCOS women; we performed comparisons using three Models. In Model A, variables were analysed with reference to healthy women with normal BMI. In Model B, variables in normal and high BMI PCOS subjects were compared to healthy women with high BMI. In Model C, the effects of BMI on PCOS subjects were analysed with reference to normal weight PCOS subjects.

Unadjusted General Linear Modelling comparing the four participant categories was performed on 20 reproductive and metabolic outcomes. These variables include clinical features (oligomenorrhea, mFG score, acne score), ovarian ultrasound findings (AFC, ovarian volume) and serum biomarkers (AMH, testosterone, ADT, DHEAS, DHT, estradiol, LH, FSH, SHBG, total cholesterol, triglycerides, HDL, LDL). HOMA-IR and FAI were also calculated and analysed. Pairwise comparison of means with Bonferroni corrections was used and mean differences (95% CI) with respective p-values were presented. Average menstrual cycle length was analysed as a dichotomous outcome, and presented as percentages with oligomenorrhea. A logistic regression was performed for this binary outcome with odds ratios (95% CI) and p-value presented. The above analyses were adjusted for socio-demographic variables. All statistical analyses were performed with the use of the Statistical Package for the Social Sciences (SPSS 25.0). Statistical significance was set at *p* < 0.05.

## Results

Of the 432 participants screened, 26 women did not meet eligibility criteria and a further 17 participants were excluded due to a lack of ovarian ultrasound data (Fig. 1). Of the remaining 389 eligible participants, 134 (34.4%) were classified as PCOS and the remaining 255 (65.6%), as the comparator healthy non-PCOS population. Overall 45.2% of women had high BMI (≥ 23). Compared to non-PCOS subjects, women with PCOS patients were younger (mean (SD): 29.84 ± 4.00 vs 32.24 ± 5.25 years, *p* < 0.001) and had higher BMI (25.14 ± 6.46 vs 23.08 ± 4.36, *p* < 0.001).

### Demographic characteristics

Table 1 displays the characteristics of women under 4 categories: non-PCOS (normal BMI), non-PCOS (high BMI), PCOS (normal BMI), and PCOS (high BMI). There were no significant differences in age comparing high and normal BMI in PCOS subjects. However in non-PCOS subjects, those with high BMI were a mean 2.21 years older. Malays and Indians had significantly higher BMI compared to those of Chinese ethnicity in those, with or without, PCOS. There were no differences in marital and employment status, monthly income, smoking habits, alcohol and coffee intake between the non-PCOS and PCOS subjects, whether of high or low BMI.

**Table 1:** Characteristics of women with, and without, PCOS according to BMI categories

BMI	Non-PCOS (n = 255)		*p* value	PCOS (n = 134)		*p* value
	Normal (< 23) n = 152	High (≥ 23) n = 103		Normal (< 23) n = 61	High (≥ 23) n = 73	
Mean age (SD)	31.35 ± 5.13	33.56 ± 5.16	0.001	29.18 ± 4.00	30.38 ± 3.94	0.083
Race n (%)			0.003			0.011
Chinese	121 (79.6)	62 (60.2)		46 (75.4)	45 (61.6)	
Malay	8 (5.3)	14 (13.6)		1 (1.6)	13 (17.8)	
Indian	7 (4.6)	13 (12.6)		4 (6.6)	7 (9.6)	
Others	16 (10.5)	14 (13.6)		10 (16.4)	8 (11.0)	
Marital status n (%)			0.039			0.379
Married	110 (72.4)	86 (83.5)		40 (65.6)	53 (72.6)	
Non-Married	42 (27.6)	17 (16.5)		21 (34.4)	20 (27.4)	
Employment status n (%)			0.475			0.194
Full time	126 (82.9)	90 (88.2)		49 (80.3)	65 (90.3)	
Part time	8 (5.3)	3 (2.9)		3 (4.9)	3 (4.2)	
Not working	18 (11.8)	9 (8.8)		9 (14.8)	4 (5.6)	
Monthly income n (%)			0.742			0.302
< $3000	70 (51.5)	52 (55.3)		19 (36.5)	34 (50.0)	
$3000-$5000	44 (32.4)	30 (31.9)		25 (48.1)	24 (35.3)	
> $5000	22 (16.2)	12 (12.8)		8 (15.4)	10 (14.7)	
Live births n (%)			0.263			0.549
Nulliparous	95 (62.9)	57 (55.9)		50 (82.0)	56 (77.8)	
Primi-/Multi-parous	56 (37.1)	45 (44.1)		11 (18.0)	16 (22.2)	
Smoking n (%)			0.926			0.109
Smoker	8 (5.3)	5 (5.0)		2 (3.4)	8 (11.6)	
Non-smoker	144 (94.7)	95 (95.0)		56 (96.6)	61 (88.4)	
Alcohol intake n (%)			0.467			0.118
Drink	64 (47.8)	35 (42.7)		30 (58.8)	24 (43.6)	
Non-drinker	70 (52.2)	47 (57.3)		21 (41.2)	31 (56.4)	
Coffee intake n (%)			0.413			0.100
Never	34 (25.4)	16 (19.5)		14 (27.5)	8 (14.5)	
Occasional	92 (68.7)	58 (70.7)		35 (68.6)	40 (72.7)	
Frequent (> 1 cup per day)	8 (6.0)	8 (9.8)		2 (3.9)	7 (12.7)	

Subjects were diagnosed as PCOS if they presented with at least 2 out of the 3 criteria, of either increased AFC ( >) 21 and/or ovarian volume (> 6.12 ml); hirsutism (mFG score ≥ 5) and/or biochemical hyperandrogenism (serum testosterone ≥ 1.89 nM); and/or oligomenorrhea (mean menstrual cycle length ≥ 35 days). Subjects were further classified as Normal (< 23) or high BMI (> 23) categories. Independent samples t-test was used for numerical variables and Pearson Chi-square test was used for categorical variables

Missing data (n): employment status (1), monthly income (25), life births (2), smoking (3), alcohol intake (39), coffee intake(39)

We utilized three models to dissect the relative effects of BMI on four categories of women (Table 2). In Model A, variables were analysed with reference to healthy-normal BMI women. In Model B, variables in normal and high BMI PCOS subjects were compared to healthy-high BMI women. In Model C, the effects of BMI on PCOS subjects were analysed with reference to PCOS women with normal weight.

**Table 2:** Effect of BMI in subjects with, and without, PCOS: Clinical and ovarian variables

Variables	Patient category	BMI	n (%)	Model A		Model B		Model C	
				Fold change	Adjusted Odds ratio (95% CI)	Fold change	Adjusted Odds ratio (95% CI)	Fold change	Adjusted Odds ratio (95% CI)
Average menstrual cycle length ≥ 35 days n (%)	Healthy	Normal	17 (11.2)	1.0	Ref				
		High	12 (11.7)	1.04	0.85 (0.32, 2.26)	1.0	Ref		
	PCOS	Normal	44 (72.1)	6.44	24.07 (9.24, 62.69)***	6.16	28.49 (8.86, 91.66)***	1.0	Ref
		High	51 (69.9)	6.24	21.25 (8.39, 53.86)***	5.97	25.15 (8.44, 74.93)***	0.97	0.88 (0.30, 2.61)

Variables	Patient category	BMI	Mean ± SD	Fold change	Adjusted Difference (95% CI)	Fold change	Adjusted Difference (95% CI)	Fold change	Adjusted Difference (95% CI)
mFG score	Healthy	Normal	1.09 ± 0.21	1.0	Ref				
		High	1.38 ± 0.25	1.27	0.06 (− 0.86, 0.98)	1.0	Ref		
	PCOS	Normal	1.75 ± 0.33	1.61	0.03 (− 1.06, 1.11)	1.27	− 0.04 (− 1.27, 1.20)	1.0	Ref
		High	3.23 ± 0.30	2.96	1.85 (0.80, 2.90)***	2.34	1.79 (0.64, 2.93)***	1.85	1.82 (0.52, 3.12)**
Acne score	Healthy	Normal	1.92 ± 0.36	1.0	Ref				
		High	1.78 ± 0.46	0.93	0.75 (− 0.63, 2.13)	1.0	Ref		
	PCOS	Normal	2.63 ± 0.58	1.37	0.13 (− 1.49, 1.74)	1.48	− 0.62 (− 2.47, 1.23)	1.0	Ref
		High	2.60 ± 0.56	1.35	1.42 (− 0.14, 2.99)	1.46	0.68 (− 1.04, 2.39)	0.99	1.30 (− 0.64, 3.23)
Mean AFC	Healthy	Normal	13.64 ± 0.78	1.0	Ref				
		High	12.65 ± 0.96	0.93	0.25 (− 2.84, 3.35)	1.0	Ref		
	PCOS	Normal	28.66 ± 1.26	2.10	12.11 (8.44, 15.78)***	2.27	11.85 (7.65, 16.06)***	1.0	Ref
		High	30.24 ± 1.17	2.22	11.34 (7.74, 14.94)***	2.39	11.08 (7.14, 15.02)***	1.06	− 0.77 (− 5.25, 3.71)
Mean OV (ml)	Healthy	Normal	4.90 ± 0.19	1.0	Ref				
		High	4.56 ± 0.23	0.93	− 0.19 (− 1.18, 0.80)	1.0	Ref		
	PCOS	Normal	8.16 ± 0.30	1.67	2.77 (1.60, 2.94)***	1.79	2.96 (1.63, 4.29)***	1.0	Ref
		High	8.55 ± 0.28	1.74	3.69 (2.54, 4.83)***	1.88	3.88 (2.53, 5.12)***	1.05	0.91 (− 0.50, 2.32)
AMH (pmol/L)	Healthy	Normal	34.88 ± 2.24	1.0	Ref				
		High	26.58 ± 2.77	0.76	− 3.04 (− 14.38, 8.30)	1.0	Ref		
	PCOS	Normal	87.97 ± 3.65	2.52	49.27 (35.67, 62.86)***	3.31	62.30 (36.81, 67.80)***	1.0	Ref
		High	75.10 ± 3.37	2.15	38.59 (25.11, 52.06)***	2.83	41.62 (26.95, 56.30)***	0.85	− 10.68 (− 27.38, 6.01)

Variables in each patient category compared against reference (Ref) denoted in each column. Model A Ref: Healthy-Normal BMI; Model B Ref: Healthy-High BMI; Model C Ref: PCOS-Normal BMI. Odds ratios and mean differences were adjusted for variables in Table 1 (except BMI).*p < 0.05; **p < 0.01; ***p < 0.001

mFG, modified Ferriman-Gallwey score; AFC, antral follicle count; OV, ovarian volume, AMH, anti-mullerian hormone

### Effect of BMI on clinical and ovarian parameters (Table 2)

As expected, about 70% of women with PCOS had oligomenorrhea. High BMI had no effect on menstrual cycle length in healthy women (Model A) while having PCOS increased the risk of oligomenorrhea 6.44-fold (Model A, OR 24.07, 95% CI 9.24–62.69). Compared to healthy women with high BMI (Model B), PCOS women whether of normal or high BMI had similar increased risks of oligomenorrhea (6.16- and 5.97-fold respectively). High BMI did not further affect rates of oligomenorrhea in women with PCOS (Model C). We were therefore unable to observe any independent effect of BMI on rates of oligomenorrhoea in healthy, or PCOS women.

In contrast, we observed a step-wise effect of BMI and PCOS on hair growth as measured by mFG scoring. About 7.6% of our cohort were hirsute as defined by mFG score ≥ 5. Compared to healthy women of normal weight (Model A), mFG scores increased by 1.27-fold in healthy women with high BMI, and by 1.61-fold in PCOS women of normal weight, although these differences did not reach statistical significance. Interestingly, women with PCOS and high BMI had a mFG score that was 2.96-fold higher compared to healthy women with normal BMI (Model A, adjusted mean difference; 1.85, 95% CI 0.80–2.90). In comparison to healthy women with high BMI (Model B), PCOS women with high, but not normal, BMI had a significantly higher mean difference in mFG (1.79, 95% CI 0.64–2.93). In women with PCOS (Model C), having high BMI also significantly increased mFG score by 1.85-fold (adjusted mean difference 1.82, 95% CI 0.52–3.12). In total, these data suggested an additive effect of high BMI and PCOS to increase mFG scores, with maximum scores observed in PCOS women with high BMI.

Although there was a trend towards higher acne scores in PCOS women, these differences did not reach statistical significance, and there was no further effect of BMI on acne scores in both healthy and PCOS women. In normal weight women, having PCOS was associated with higher mean adjusted differences in AFC (12.11, 95% CI 8.44–15.78), ovarian volume (2.77, 95% CI 1.60–2.94) mls, and AMH (49.27, 95% CI 35.67–62.86) pmol/L compared to healthy women (Model A). However, there was no additional effect of BMI on mean AFC, mean ovarian volume and AMH in both healthy and PCOS women (Models A, B, C).

### Effect of BMI on reproductive hormones (Table 3)

**Table 3:** Effect of BMI in subjects with, and without, PCOS: Reproductive hormones

Variables	Patient category	BMI	Mean ± SD	Model A		Model B		Model C	
				Fold change	Adjusted difference (95% CI)	Fold change	Adjusted difference (95% CI)	Fold change	Adjusted difference (95% CI)
Testosterone (nmol/L)	Healthy	Normal	1.10 ± 0.05	1.0	Ref				
		High	1.12 ± 0.06	1.02	0.12 (− 0.12, 0.36)	1.0	Ref		
	PCOS	Normal	2.05 ± 0.08	1.86	0.86 (0.58, 1.15)***	1.83	0.74 (0.42, 1.07)***	1.0	Ref
		High	2.06 ± 0.07	1.87	0.93 (0.66, 1.21)***	1.84	0.82 (0.52, 1.12)***	1.0	0.07 (− 0.27, 0.41)
ADT (nmol/L)	Healthy	Normal	7.77 ± 0.49	1.0	Ref				
		High	6.82 ± 0.59	0.88	− 0.01 (− 2.78, 2.76)	1.0	Ref		
	PCOS	Normal	11.64 ± 0.77	1.50	4.17 (0.91, 7.43)**	1.71	4.18 (0.47, 7.89)*	1.0	Ref
		High	10.08 ± 0.71	1.30	2.40 (− 0.76, 5.57)	1.48	2.41 (− 1.03, 5.85)	0.87	− 1.77 (− 5.67, 2.14)
DHEAS (µmol/L)	Healthy	Normal	5.28 ± 0.18	1.0	Ref				
		High	4.93 ± 0.22	0.93	0.12 (− 0.80, 1.04)	1.0	Ref		
	PCOS	Normal	5.95 ± 0.29	1.13	0.76 (− 0.33, 1.84)	1.21	0.64 (− 0.60, 1.88)	1.0	Ref
		High	6.23 ± 0.26	1.18	1.24 (0.19, 2.29)*	1.26	1.12 (− 0.03, 2.27)	1.05	0.48 (− 0.82, 1.78)
DHT (nmol/L)	Healthy	Normal	1.22 ± 0.08	1.0	Ref				
		High	1.38 ± 0.10	1.13	0.28 (− 0.19, 0.75)	1.0	Ref		
	PCOS	Normal	1.49 ± 0.13	1.22	0.13 (− 0.42, 0.68)	1.22	− 0.15 (− 0.78, 0.48)	1.0	Ref
		High	1.84 ± 0.12	1.51	0.73 (0.19, 1.26)**	1.33	0.45 (− 0.14, 1.03)	1.23	0.59 (− 0.07, 1.25)
SHBG (nmol/L)	Healthy	Normal	67.36 ± 2.18	1.0	Ref				
		High	43. 56 ± 2.65	0.65	− 23.36 (− 34.59, − 12.14)**	1.0	Ref		
	PCOS	Normal	61.34 ± 3.44	0.91	− 9.08 (− 22.29, 4.14)	1.41	14.29 (− 0.80, 29.37)	1.0	Ref
		High	32.04 ± 3.14	0.48	− 32.91 (− 45.73, − 20.08)***	0.74	− 9.54 (− 23.51, 4.43)	0.52	− 23.83 (− 39.68, − 7.98)***
FAI	Healthy	Normal	2.03 ± 0.29	1.0	Ref				
		High	3.48 ± 0.35	1.71	1.70^*^(0.21, 3.20)*	1.0	Ref		
	PCOS	Normal	4.19 ± 0.45	2.06	2.04 (0.30, 3.79)*	1.20	0.34 (− 1.66, 2.34)	1.0	Ref
		High	9.45 ± 0.41	4.66	6.97 (5.28, 8.67)***	2.72	5.27 (3.42, 7.12)***	2.26	4.93 (2.84, 7.02)***
LH (IUL)	Healthy	Normal	4.74 ± 0.33	1.0	Ref				
		High	4.04 ± 0.39	0.85	− 0.77 (− 2.42, 0.87)	1.0	Ref		
	PCOS	Normal	9.13 ± 0.49	1.93	4.61 (2.69, 6.52)***	2.26	5.38 (3.22, 7.54)***	1.0	Ref
		High	8.11 ± 0.45	1.71	2.02 (0.18, 3.86)*	2.01	2.79 (0.80, 4.79)**	0.89	− 2.58 (− 4.83, − 0.34)*
FSH (IUL)	Healthy	Normal	7.94 ± 0.19	1.0	Ref				
		High	6.88 ± 0.30	0.87	− 0.62 (− 1.61, 0.37)	1.0	Ref		
	PCOS	Normal	7.67 ± 0.23	0.97	− 0.69 (− 1.85, 0.47)	1.11	0.07 (− 1.40, 1.26)	1.0	Ref
		High	6.29 ± 0.28	0.79	− 1.62 (− 2.75, − 0.49)**	0.91	1.00 (− 2.23, 0.23)	0.82	− 0.93 (− 2.32, 0.46)
Estradiol (pmol/L)	Healthy	Normal	175.03 ± 5.22	1.0	Ref				
		High	172.96 ± 6.28	0.99	2.26 (− 28.02, 32.53)	1.0	Ref		
	PCOS	Normal	168.85 ± 8.22	0.96	− 1.67 (− 37.73, 34.40)	0.98	− 3.92 (− 44.88, 37.03)	1.0	Ref
		High	190.70 ± 7.46	1.09	23.18 (− 11.42, 57.79)	1.10	20.92 (− 16.67, 58.51)	1.13	24.85 (− 18.02, 67.71)

ADT, androstenedione; DHEAS, dehydroepiandrosterone sulphate; DHT, dihydrotestosterone; SHBG, sex hormone binding globulin; FAI, free androgen index; LH, luteinizing hormone; FSH, Follicle stimulating hormone

Compared to their healthy normal BMI counterparts (Model A), fasting serum testosterone levels were 1.86- to 1.87-fold higher in PCOS women of normal and high BMI respectively. When the reference was healthy-high BMI women (Model B), PCOS women whether of normal or high BMI exhibited similarly increased testosterone levels of 1.83- and 1.84-fold respectively. High BMI did not change testosterone levels amongst PCOS women (Model C). In healthy women (Model A), an increase in BMI did not change levels of ADT, DHEAS and DHT. PCOS women with high BMI had higher adjusted mean differences in DHEAS (1.24, 95% CI 0.19–2.29) μMol/L and DHT (0.73, 95% CI 0.19–1.26) nMol/L compared to healthy women with normal BMI (Model A). Like testosterone, these differences were no longer observed when the references were healthy-high BMI (Model B) or PCOS-normal BMI women (Model C).

As expected, high BMI significantly *decreased* SHBG levels by 35% and 52% in healthy and PCOS women respectively (Model A). PCOS status did not have an independent effect on SHBG levels (Model B). Since decreases in SHBG directly increases FAI, healthy women with high BMI had higher FAI levels (Model A). Compared to healthy women with high BMI (Model B), PCOS in the absence of high BMI did not affect FAI levels. The combined effect of PCOS and high BMI, however, resulted in FAI increasing by 2.72-fold (adjusted mean difference 5.27, 95% CI 3.42–7.12). In PCOS women (Model C), high BMI also increased FAI by 2.26-fold (adjusted mean difference 4.93, 95% CI 2.84–7.02). In total, BMI and PCOS had an additive effect to increase FAI levels, with the highest FAI observed in PCOS women of high BMI.

Compared to healthy-normal BMI women (Model A), LH levels were 1.93- and 1.71-fold higher in PCOS women of normal weight and high BMI respectively. Likewise, PCOS women of normal weight and high BMI had a 2.26- and 2.01-fold increase in LH levels respectively when compared to healthy women with high BMI (Model B). While LH levels were higher in PCOS women when compared to their healthy non-PCOS counterparts, we observed that a high BMI significantly lowered LH levels amongst high BMI PCOS women when compared to PCOS women of normal weight (Model C, adjusted mean difference − 2.58, 95% CI − 24.83, − 0.34).

Similarly, an increase in BMI did not change FSH levels in healthy women (Model A). PCOS women with high BMI had lower adjusted mean difference in FSH (− 1.62, 95% CI − 2.75, − 0.49) when compared to healthy women of normal weight (Model A). However, no effect of BMI on FSH levels was observed when compared to normal weight PCOS women (Model C). Estradiol was not affected by weight in both healthy (Model A) and PCOS women (Model C).

### Effect of BMI on metabolic biomarkers (Table 4)

**Table 4:** Effect of BMI in subjects with, and without, PCOS: Metabolic parameters

Variables	Patient category	BMI	Mean ± SD	Model A		Model B		Model C	
				Fold change	Adjusted difference (95% CI)	Fold change	Adjusted difference (95% CI)	Fold change	Adjusted difference (95% CI)
HOMA-IR	Healthy	Normal	1.29 ± 0.15	1.0	Ref				
		High	2.20 ± 0.19	1.71	0.68 (0.01, 1.35)*	1.0	Ref		
	PCOS	Normal	1.23 ± 0.24	0.95	0.05 (− 0.74, 0.84)	0.56	− 0.63 (− 1.53, 0.27)	1.0	Ref
		High	3.53 ± 0.22	2.74	1.48 (0.71. 2.25)***	1.60	0.80 (− 0.03, 1.64)	2.87	1.43 (0.48, 2.38)***
Cholesterol (mmol/L)	Healthy	Normal	4.63 ± 0.07	1.0	Ref				
		High	5.10 ± 0.08	1.10	0.42 (0.09, 0.74)**	1.0	Ref		
	PCOS	Normal	4.71 ± 0.11	1.02	0.09 (− 0.30, 0.48)	0.92	− 0.33 (− 0.77, 0.11)	1.0	Ref
		High	4.96 ± 0.10	1.07	0.16 (− 0.21, 0.54)	0.97	− 0.25 (− 0.66, 0.16)	1.05	0.08 (− 0.39, 0.54)
Triglycerides (mmol/L)	Healthy	Normal	0.80 ± 0.06	1.0	Ref				
		High	1.30 ± 0.07	1.63	0.35 (0.15, 0.55)***	1.0	Ref		
	PCOS	Normal	0.81 ± 0.09	1.01	0.03 (− 0.21, 0.27)	0.62	− 0.32 (− 0.59, − 0.05)*	1.0	Ref
		High	1.24 ± 0.08	1.55	0.36 (0.13, 0.59)***	0.95	0.01 (− 0.24, 0.26)	1.53	0.33 (0.04, 0.61)*
HDL (mmol/L)	Healthy	Normal	1.59 ± 0.03	1.0	Ref				
		High	1.35 ± 0.03	0.85	− 0.21 (− 0.36, − 0.07)***	1.0	Ref		
	PCOS	Normal	1.63 ± 0.04	1.03	0.00 (− 0.17, 0.17)	1.21	0.21 (0.01, 0.40)*	1.0	Ref
		High	1.28 ± 0.04	0.81	− 0.33 (− 0.50, − 0.17)***	0.95	− 0.12 (− 0.30, 0.06)	0.74	− 0.33 (− 0.53, − 0.12)***
LDL (mmol/L)	Healthy	Normal	2.66 ± 0.06	1.0	Ref				
		High	3.20 ± 0.08	1.20	0.45 (0.17, 0.74)***	1.0	Ref		
	PCOS	Normal	2.72 ± 0.10	1.02	0.15 (− 0.19, 0.49)	0.85	− 0.30 (− 0.69, 0.08)	1.0	Ref
		High	3.12 ± 0.09	1.18	0.37 (0.04, 0.70)*	0.98	− 0.08 (− 0.44, 0.27)	1.15	0.22 (− 0.19, 0.62)

HOMA‐IR, homoeostatic model assessment‐insulin resistance; HDL, high-density lipoprotein; LDL, low-density lipoprotein

Unsurprisingly in healthy women (Model A), high BMI worsened HOMA-IR, cholesterol, triglycerides, LDL and HDL levels. High BMI increased HOMA-IR by 1.71-fold (adjusted mean difference 0.68, 95% CI 0.01–1.35) in healthy women (Model A), and by 2.87-fold (adjusted mean difference 1.43, 95% CI 0.48–2.38) in PCOS women (Model C). Although high BMI increased insulin resistance by 1.60-fold in PCOS women when the reference group was healthy women with high BMI (Model B), this increase of 0.80 did not reach statistical significance. Increased BMI was associated with higher HOMA-IR, triglycerides and lower HDL in the PCOS group (Model C).

## Discussion

Our study involving both healthy and PCOS women, revealed hitherto underappreciated insights into the effects of BMI and PCOS on a broad spectrum of clinical, hormonal and metabolic characteristics commonly associated with the syndrome. We observed an additive effect of BMI and PCOS to increase hair growth, with maximum mFG score observed in PCOS women with high BMI. This effect was mirrored by the additive effect of BMI and PCOS on serum levels of free androgen as reflected by FAI. However, we were unable to observe any independent effect of high BMI on rates of oligomenorrhoea, AFC, ovarian volume and serum androgen levels in healthy or PCOS women.

Increased hair growth, as reflected by mFG scoring, was worsened by the combined effects of high BMI and PCOS. Having PCOS increased mFG scores in women with high BMI (Model B) by 2.34-fold (adjusted mean difference 1.85, 95% CI 0.80–2.90) while having high BMI independently increased mFG scores in women with PCOS (Model C) by 1.85-fold (adjusted mean difference 1.82, 95% CI 0.52–3.12). An increase in mFG score of 1.82, may represent a group of women whose hair growth is out of the ‘norm’ and be cosmetically important in less hirsute East Asian societies [19]. Such increases in mFG score may not be trivial and can affect the self-esteem of women with PCOS [1], further adding to any insecurities and emotional distress they might already face from being overweight or obese [1–3]. This quantum increase in mFG score may also have significance for diagnosis of PCOS since there are proposals for mFG cut-off score to be lowered to 3, instead of 8, for Far East and South East Asian women [20].

Increased mFG scores were not associated directly with increased androgens per se, but rather with raised FAI, an index of bioavailable testosterone, underscoring the well-known effects of high BMI to lower the androgen-binding protein, SHBG [16]. It is relevant to note that compared to normal weight healthy women, PCOS women with high BMI have 1.51-fold increase in DHT levels, perhaps contributing to a direct effect on hair follicles through the androgen receptor [17]. Although a cause and effect association cannot be deduced from this cross-sectional study, the additive effects of BMI and PCOS status to increase mFG score merit further study. Is weight reduction then a possible therapy for hairiness? In a systemic review, weight loss following lifestyle modification reduced total testosterone and hirsutism [21]. Furthermore, a meta-analysis indicated that weight loss following bariatric surgery was associated with reduced hirsutism, and decreased serum free testosterone levels [22]. Since the insulin sensitizing agent metformin has been reported to decrease BMI and testosterone levels in PCOS women [23], its application to reduce hirsutism merits further exploration. The challenge of the future is to devise sustainable measures to achieve optimal weight and examine its impact on hirsutism.

We did not observe any effect of BMI on rates of oligomenorrhea in healthy and PCOS women. The lack of effect of BMI on oligomenorrhea could be because mean BMI in our cohort were in overweight (PCOS: 25.14 ± 6.46; non-PCOS: 23.08 ± 4.36), rather than the obese range (> 27.5). Similarly severity of oligomenorrhea was not affected by BMI in a Korean cohort [24] where the average BMI was similar to our cohort (PCOS: 24.4 ± 4.6; Non-PCOS: 21.4 ± 2 ± 6). In contrast, BMI in the obese range of 31.2 ± 4.4 in a Taiwanese PCOS cohort was reported to prolong menstrual intervals [25]. Indeed in severely obese subjects, weight loss due to lifestyle interventions [26] or bariatric surgery [22] were reported to improve menstrual regularity. Whether severe obesity in Singaporean women will affect menstrual cycle length needs to be determined in a larger study.

We also did not observe any effects of BMI on the ovarian parameters, AFC and ovarian volume, in both PCOS and healthy women, consistent with other studies [27]. There have been conflicting results on the relationship between BMI and AMH. A meta-analysis found that AMH concentrations are significantly lower in obese women, regardless of PCOS status [28]. We did observe a similar trend, not reaching statistical significance, in both healthy and PCOS groups. Interestingly, a study has found that elevated BMI correlates negatively with AMH in Caucasian women but this effect was not observed in African-American, Hispanic or Asian women [29]. Nevertheless, further clinical studies would be needed to further explore interaction of race, obesity and ovarian reserve as measured by AMH.

As expected, increased BMI was associated with poorer metabolic status in both groups, with insulin resistance and deranged lipid profiles more common in high BMI PCOS patients. Compared to normal weight healthy women (Model A), women with PCOS and high BMI showed a 2.74-fold increase in HOMA-IR. In women with high BMI (Model B), having PCOS increased HOMA-IR by 1.60-fold. Although this difference did not reach statistical significance, the trends are consistent with well- established findings that PCOS can worsen metabolic status in obese patients [13]. Interestingly, a positive relationship between insulin resistance and hyperandrogenism has been reported previously [30]. Insulin resistance and associated hyperinsulinemia enhances ovarian steroidogenesis, thereby resulting in increased androgen levels and related hyperandrogenic features [31]. Our observation that obesity accentuates insulin resistance in PCOS subjects could further account for the effect of obesity on clinical hyperandrogenism, as defined by mFG score.

A possible limitation of our study would be the increased risk of type 1 error since multiple comparisons between the four categories across all twenty variables were performed. However, Bonferroni correction was applied to control for the occurrence of false positives and counteract the problem of multiple comparisons. We also used BMI ≥ 23 as the cut-off, thereby combining overweight (BMI ≥ 23 to < 25) and obese (BMI ≥ 25) categories, possibly masking effects that may become evident only with obesity. For example, there is evidence suggesting that only PCOS women who were obese, but not those merely overweight, exhibited raised total testosterone [13]. For future research with a larger sample size, high BMI subjects could be subclassified into overweight and obese categories for further analysis. We acknowledge that a certain proportion of women have undergone hair removal procedures. However, this is unlikely to bias our results as there is little reason for hair removal procedures to be more common in any of our reference groups.

A strength of our study was the use of two separate study groups, healthy non-PCOS subjects who were recruited from an annual health screening and PCOS subjects referred from the two tertiary gynaecological referral clinics in Singapore. While there have been several studies on the effect of obesity on the reproductive and metabolic outcomes of PCOS [13], few have compared the differential effect of weight on outcomes in both PCOS and healthy women. Comparison between the two groups allowed for a comprehensive evaluation of the effects of BMI on various outcomes and if these effects are exacerbated by PCOS.

## Conclusion

Our study examined the effects of increased BMI on the three diagnostic features of PCOS; hyperandrogenism, defined clinically and/or biochemically, oligomenorrhea and polycystic ovarian morphology, as well as its effect on other metabolic outcomes. This is especially relevant given that obesity is less prevalent in East Asian women with PCOS [29]. We observed for the first time that being overweight or obese had an additive effect to significantly increase mFG scores in PCOS women. This was associated with a decrease in SHBG and consequently an increase in FAI. Our findings align with current recommendations of implementing lifestyle interventions as the first-line non-pharmacological treatment for PCOS. Lifestyle interventions in diet and physical activity have been associated with improvements in hirsutism [30], menstrual abnormalities [26, 31], ovarian dysmorphology [31] as well as improvements in hormonal and metabolic parameters [21].

Understanding the exacerbating effect on BMI on various parameters such as hirsutism and insulin resistance emphasizes the importance of managing obesity in the treatment of PCOS symptoms as well as its co-morbidities. Clinical trials are required to examine the effects of optimizing BMI to improve hirsutism and to manage the metabolic derangements observed in PCOS patients.

## Data Availability

The data that support the findings of this study are available from the corresponding author upon reasonable request, subject to institutional and ethical board approvals.

## Abbreviations

PCOS: Polycystic Ovary Syndrome
BMI: Body Mass Index
AFC: Antral follicle counts
mFG: Modified Ferriman-Gallwey score
NUH: National University Hospital
AMH: Anti-müllerian hormone
FSH: Follicle stimulating hormone
ADT: Androstenedione
DHEAS: Dehydroepiandrosterone sulphate
DHT: Dihydrotestosterone
SHBG: Sex hormone binding globulin
LH: Luteinizing hormone
HDL: High-density lipoprotein
LDL: Low-density lipoprotein
HOMA-IR: Homeostasis model assessment‐estimated insulin resistance
FAI: Free androgen index
